# Overexpression of *POLQ* Confers a Poor Prognosis in Early Breast Cancer Patients

**DOI:** 10.18632/oncotarget.124

**Published:** 2010-07-09

**Authors:** Geoff S Higgins, Adrian L Harris, Remko Prevo, Thomas Helleday, W Gillies McKenna, Francesca M Buffa

**Affiliations:** ^1^Gray Institute for Radiation Oncology and Biology; ^2^Molecular Oncology Laboratories, University of Oxford, UK

**Keywords:** Translational research, POLQ, breast cancer, prognosis, radiotherapy

## Abstract

Depletion of POLQ (DNA polymerase theta) has recently been shown to render tumour cells more sensitive to radiotherapy whilst having little or no effect on normal tissues. This finding led us to investigate whether tumours that overexpress *POLQ* are associated with an adverse outcome. We therefore correlated the clinical outcomes of two retrospective series of patients with early breast cancer with the expression levels of *POLQ*, as determined by microarray gene expression analysis. We found that a significant number of tumours overexpressed *POLQ* and that overexpression was correlated with ER negative disease (p=0.047) and high tumour grade (p=0.004), both of which are associated with poor clinical outcomes. *POLQ* overexpression was associated with poor relapse free survival rates on both univariate (HR 5.80; 95% CI, 2.220 to 15.159; p<0.001) and multivariate analysis (HR 8.086; 95% CI 2.340 to 27.948 p=0.001). Analysis of other published clinical series confirmed that *POLQ* overexpression is associated with adverse clinical outcomes. The poor prognosis associated with *POLQ* is independent of other clinical or pathological features. The mechanism that causes this adverse outcome remains to be elucidated but may in part arise from resistance to adjuvant treatment. These findings, combined with the limited normal tissue expression of POLQ, make it a very appealing target for possible clinical exploitation.

## INTRODUCTION

POLQ (DNA Polymerase Theta) is a member of the A family of DNA polymerases, which, unusually for this class of polymerases, synthesizes DNA with very low fidelity [1, 2]. The precise physiological functions of this protein are currently unclear. It has previously been suggested that mice deficient in POLQ had a substantially decreased frequency of mutations in immunoglobulin genes [3, 4]. However a recent study found that mutation types and frequencies were similar in wild type, *POLQ*−/−, *POLH*−/−, and *POLQ*−/− *POLH*−/− mice [5]. Accordingly this group suggested that POLQ does not have a significant role in the hypermutation pathway.

It has been suggested that POLQ has a role in base excision repair (BER) but this also remains unresolved. It has previously been shown in the DT40 chicken B cell lymphoma line, that *POLQ*/*POLβ* mutants had significantly higher sensitivity to methyl methanesulfonate than either single mutant. Extracts obtained from this cell line were used to show that *POLQ* mutant cells have markedly reduced single nucleotide BER capacity in vitro and that this reduction was of a similar magnitude to cells deficient in *POLβ* [6]. These findings led to the suggestion that POLQ and POLβ cooperate in BER.

Recent biochemical analysis has shown that cloned full-length human POLQ as well as a C-terminal fragment of POLQ, have 5'-deoxyribose phosphate (5'- dRP) lyase activity. The full-length protein and the C-terminal fragment were shown to have BER activity in vitro [7]. Although these findings have been used to support the argument that POLQ may have a role in BER in vivo, it should be noted that the rate of 5'-dRP lyase activity of POLQ is approximately 40 fold slower than that of POLβ. Cells with deficiencies in the BER pathway have been shown to have increased sensitivity to temozolomide [8]. Since cells depleted of POLQ do not show hypersensitivity to this drug, it has been questioned as to whether POLQ has any physiologically significant role in BER [9].

We have recently published a siRNA screen that aimed to identify molecular determinants of tumour radiosensitivity [9]. This study demonstrated that *POLQ* siRNA transfection resulted in radiosensitisation of a panel of tumour cell lines but had little or no effect on normal tissue lines. These differences reflect previous work showing significant disparity in expression between normal tissues and tumour cells [10]. Normal tissue expression appears to be mainly limited to lymphoid tissues such as the fetal liver, thymus, and bone marrow. However POLQ is known to be overexpressed in a large proportion of tumours derived from patients with colon, lung, and gastric cancer.

In view of the in vitro evidence linking *POLQ* expression to tumour cell radioresistance, we hypothesised that *POLQ* overexpression may increase the likelihood of treatment failure in cancer patients, and therefore confer an adverse clinical prognosis.

We therefore correlated the clinical outcomes of two series of breast cancer patients (n=279 in total) with the expression levels of *POLQ* as determined by microarray gene expression analysis. We also analysed the pathways associated with *POLQ* expression in vivo by data-mining gene expression data from published breast cancer studies (n=1015 samples). To the best of our knowledge this is the first study to demonstrate that *POLQ* is overexpressed in breast cancer, that its overexpression confers a significant adverse prognosis, and that it is associated with key cancer pathways.

## MATERIALS AND METHODS

### Ethics Statement

Informed consent was obtained and all clinical investigations were conducted according to the ethical standards and principles expressed in the Declaration of Helsinki. Ethical approval was obtained from the local research ethics committee.

### Patient Details

Individual tumour samples were obtained from retrospective series of patients with early primary breast cancer who were treated in Oxford, UK, between 1989 and 1998. Patients received adjuvant chemotherapy and/or adjuvant hormone therapy, or no adjuvant treatment. Tamoxifen was used as endocrine therapy for 5 years in estrogen receptor (ER) positive patients. Patients who were ≥50 years of age, with lymph node positive tumors, or ER– and/or a primary tumor >3 cm in diameter, received adjuvant cyclophosphamide, methotrexate, and 5-fluorouracil (CMF) for six cycles, in a three weekly intravenous regimen. Patients ≥50 years of age with ER–, lymph node–positive tumors also received CMF. Two series of 152 (Series 1) and 127 (Series 2) samples respectively were analysed. Series 1 has been described previously [11]; this series had completed 7 years of follow-up for all but 4 patients, and the median follow-up time for patients leaving the study alive and without a relapse was 12 years. Series 2 is part of a published series [12]; the published cohort had 93 cases in common with Series 1, these have been excluded from this study so that Series 1 and 2 have no overlapping cases. Series 2 had completed 10 year of follow-up apart from one case. Patient demographic details of Series 1 and 2 as analysed in this study are summarised in supplementary table 1.

### RNA Extraction and Gene Expression Profiling

Total RNA was isolated by Trizol method (Invitrogen, Carlsbad, CA) according to the manufacturer's instructions. mRNA expression was measured using Affymetrix U133 arrays for Series 1 and Illumina Human RefSeq-8 arrays (Illumina inc., San Diego, CA, USA) for Series 2. RNA was amplified using Ambion Illumina Amplification Kit. Methods for both protocols have been previously described [12, 13]. Affymetrix data were pre-processed using gcrma [14]; signal from Illumina arrays was background subtracted with local background subtraction (BeadStudio). Data from both series were quantile normalized in Bioconductor (www.bioconductor.org) and logged (base 2). The target sequence of the probes that corresponded to POLQ expression in Affymetrix and Illumina arrays are shown in supplementary table 2. Two additional published datasets of patients with early breast cancer were accessed to validate the findings observed in the Oxford datasets [15, 16].

### Published Clinical Series

NCBI Gene Expression Omnibus (http://www.ncbi.nlm.nih.gov/geo/) was searched for gene expression studies in cancer, published in peer-reviewed journals, where microarrays were performed on frozen material extracted before treatment with either chemotherapy, radiotherapy or endocrine treatment. Five data sets [11, 15, 17] of 1015 samples in total (supplementary table 3) were selected that used latest generation Affymetrix 3' array platforms (Affymetrix U133 and plus2, www.affymetrix.com). All handling and processing of the downloaded data was performed as previously described [18].

### Data-mining of Gene Expression Data

Seed-clustering with bootstrap resampling was applied as previously described [18] to obtain genes co- and inversely expressed with POLQ in the 1015 published breast cancer samples. In short, the two probesets targeting POLQ (supplementary table 2) were chosen as initial seeds. Transcripts on the arrays showing significant association (Spearman Rank Test, Bonferroni multiple test correction) with each seed after bootstrap resampling of the breast cancer samples were considered. Amongst these, transcripts showing a concordant association with both seeds that was significantly higher than observed by random simulation were selected as POLQ co-/inversely expressed genes. A pathway enrichment analysis was thus performed using GeneCodis2 [19] to study the Gene Ontology classes and the KEGG pathways which are over-represented in POLQ co-/inversely expressed genes.

### Survival Analysis

Endpoints were relapse free survival for Series 1; and distant-relapse free survival and recurrence free survival as defined by the STEEP criteria [20] for Series 2. Endpoints as published were considered for the other datasets. Univariate and multivariate analysis was performed. Cox multivariate models were reduced using stepwise backward likelihood selection. In univariate analyses, expression of POLQ and other genes was considered either as binary variable, with median expression as binary cut-off, or as continuous variable, ranked and normalised between 0 and 1. In multivariate analysis the latter was always considered.

## RESULTS

### *POLQ* is overexpressed in breast cancer compared to normal breast tissue

**Fig 1:**
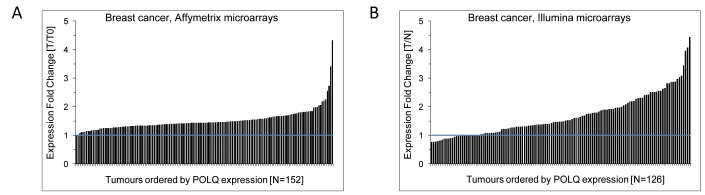
*POLQ* Expression in Breast Cancer A) Breast cancer samples, Series 1, described in this study (N=152). No normal breast tissue samples were available for Series 1 so *POLQ* data were normalised to the sample with the lowest expression of *POLQ* (named T0). Expression fold change (FC) between all other tumours and T0 is shown for *POLQ* (207746_at). Expression is measured by Affymetrix array and quantile normalized. B) Breast cancer samples, Series 2, (N=127) described in this study. The FC between *POLQ* (ILMN_1450687) expression in each tumour and the median expression of 10 normal pools is shown. Expression is measured by Illumina arrays and quantile normalized.

In order to assess *POLQ* expression, we identified two independent gene expression datasets that were obtained using arrays from different manufacturers. Series 1 and 2 were obtained using Affymetrix and Illumina arrays respectively. *POLQ* expression was normalised to the lowest level of tumour expression in the Affymetrix series, and to a panel of normal breast tissue samples for the Illumina series. *POLQ* expression is upregulated in a large proportion of breast tumour samples (Fig 1).

### *POLQ* overexpression is independently associated with significantly worse relapse free survival (RFS) rates

**Fig. 2:**
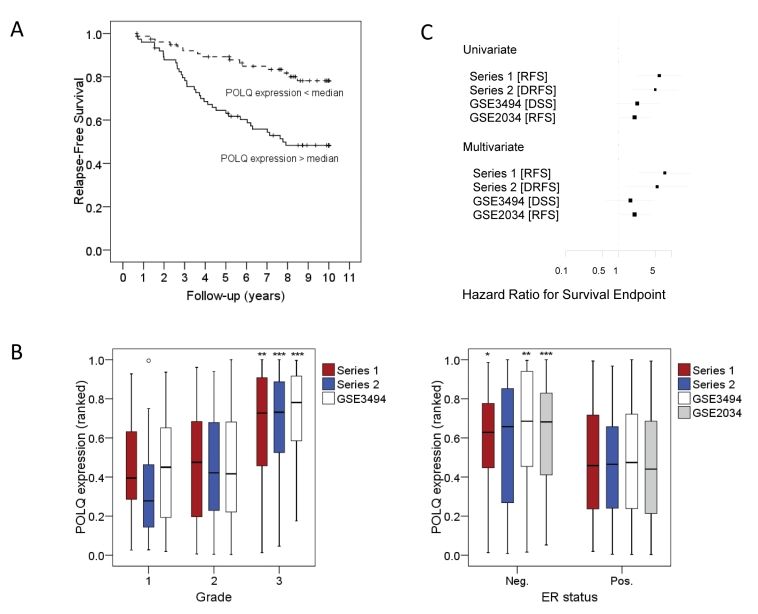
*POLQ* expression is prognostic in breast cancer independently from clinico-pathological variables. A) Univariate analysis in 152 breast cancers (Series 1). *POLQ* expression is divided in two groups by median value. B) *POLQ* expression is associated with tumour grade (left) and ER status (right) in Series 1 and 2 described in this study (Affymetrix and Illumina arrays respectively) and two published series (Affymetrix arrays, see Methods), although grade information was not available for GSE2034. Boxes summarize the median, quartiles and extreme values of *POLQ* expression in the different categories. One outlier is shown (circle), defined as case with values between 1.5-3 box lengths from the edge of the box. Mann-Whitney and Spearman Rank Association significance levels for the null hypotheses of *POLQ* expression not varying with ER and Grade respectively, are indicated on the highest category of each plot:*=p<0.05, **=p<0.01, ***=p<0.001. C) Forest plot of *POLQ* Hazard Ratio for Survival Endpoints in univariate and multivariate analysis in the 2 series described in this study and 2 published datasets (GEO Ids shown). Dots represent Hazard Ratios of *POLQ* expression and grey bars the 95% confidence intervals. Dot dimensions are proportional to dataset size. The expression of *POLQ* is entered in this model as a continuous ranked variable, normalised between 0 (lowest rank) and 1 (highest rank). RFS= Recurrence Free Survival, DRFS=Distant Relapse Free Survival, DSS= Disease Specific Survival.

The samples from Series 1 were divided into the top and bottom 50th centiles and a univariate analysis of the differences in RFS was conducted (Fig 2A). *POLQ* overexpression was associated with a markedly increased risk of disease relapse (HR 5.80; 95% CI, 2.220 to 15.159; p<0.001). We then correlated the level of *POLQ* expression with multiple pathological and demographic features such as patient age, tumour grade and tumour size. We found that *POLQ* overexpression correlated with both ER negative disease (p=0.047) and high tumour grade (p=0.004) (Fig 2B). As both of these features are recognised as being associated with poor clinical outcomes [21-23], we performed a multivariate analysis which showed that *POLQ* expression confers a poor prognosis which is independent of any other clinical features (HR 8.086; 95% CI 2.340 to 27.948; p=0.001). The multivariate models contained *POLQ* as continuous variable, ranked and normalised between 0 and 1, and the following clinical features; ER status, lymph node status, patient age, tumour grade, tumour size. To confirm the validity of this finding we performed further univariate and multivariate analyses on Series 2 and the two additional datasets previously described (supplementary table 4). In total, three of the four datasets analysed demonstrated that *POLQ* overexpression was strongly associated with significantly worse survival outcomes (Fig 2C).

### Clustering analysis identifies genes co-expressed with *POLQ* with functions in key cancer pathways

**Fig. 3:**
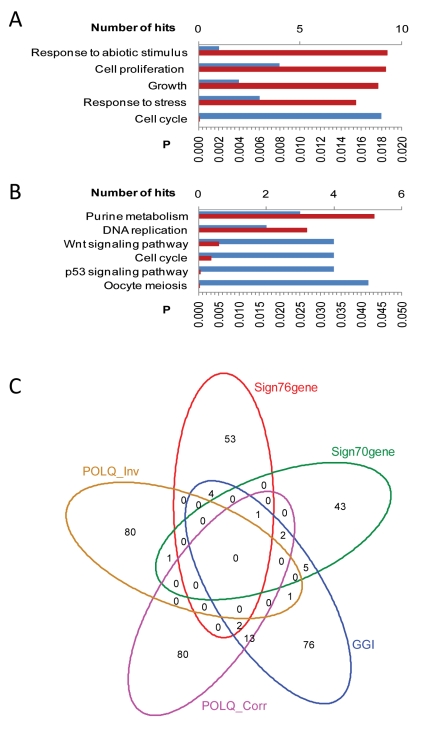
Pathway analysis and overlap with prognostic signatures of *POLQ* co-expressed genes Seed-clustering was used in 1015 breast cancer samples to identify genes whose expression was co and inversely associated with *POLQ* expression. A) Over-represented KEGG pathways and B) GO Biological processes amongst genes co-expressed with *POLQ*. The number of genes in each pathway is shown in blue, top x-axis, and a hypergeometric test p-value (FDR adjustment for multiple testing) is shown in red, bottom axis. C) Venn-diagram showing the overlap of genes whose expression is co- (POLQ_Corr) and inversely (POLQ_Inv) associated with expression of *POLQ* with the Genomic Grade Index Signature (GGI) [25], the 76-gene signature (Sign76gene) [16], and the 70- genes signature (Sign70genes) [24].

In order to identify genes which were co-expressed with *POLQ*, a seed-clustering analysis was performed on gene expression data obtained from five different breast cancer data sets (details of datasets in supplementary table 3). This identified a total of 97 genes that were strongly associated with *POLQ* overexpression in breast cancer (supplementary table 5). Pathway analysis of these genes showed that genes co-expressed with *POLQ* are involved in several pathways that have been associated with cancer development and progression such as cell cycle progression, p53 signalling, Wnt signalling and DNA replication (Fig 3A and 3B).

### Genes co-expressed with *POLQ* overlap with several genes that comprise the Gene expression Grade Index (GGI)

Previous studies such as the ‘70-gene’ expression signature [24] have identified groups of genes that form expression profiles which correlate with clinical outcome. Although *POLQ* expression has not previously been shown to be independently associated with clinical outcome, it is interesting to note that *POLQ* is included in both the GGI [25], and the '76- gene' signature [16]. The correlation between *POLQ* expression and tumour grade and prognosis (Fig. 2) led us to assess whether genes that are co-expressed with *POLQ* are included in these validated gene expression signatures (Fig. 3C). Eighteen of the genes that are significantly co-expressed with *POLQ* (supplementary table 5) are components of the GGI index (Table 1). The large number of genes that overlap between these two groups may account for the clinical correlation between *POLQ* expression and high tumour grade.

**Table 1:** Overlap between the Genomic Grade Index (GGI) signature [25] and transcripts co- or inversely associated with *POLQ* in seed-clustering of 1015 breast cancer samples

Symbol	GGI grade^s^	Accession Number	Gene ID	Full name/description
Transcripts co-expressed with POLQ				
AURKA	G_3_	NM_003158	6790	aurora kinase A
CCNB2	G_3_	NM_004701	9133	cyclin B2
CCNB2	G_3_	NM_004701	9133	cyclin B2
CCNE2	G_3_	NM_004702	9134	cyclin E2
CDKN3	G_3_	AF213033	1033	cyclin-dependent kinase inhibitor 3 (CDK2 associated dual specificity phosphatase)
CEP55	G_3_	NM_018131	55165	centrosomal protein 55kDa
ESPL1	G_3_	NM_012291	9700	extra spindle pole bodies homolog 1 (S. cerevisiae)
ESPL1	G_3_	D79987	9700	extra spindle pole bodies homolog 1 (S. cerevisiae)
GTSE1	G_3_	NM_016426	51512	G-2 and S-phase expressed 1
KIFC1	G_3_	BC000712	3833	kinesin family member C1
LMNB1	G_3_	NM_005573	4001	lamin B1
MCM2	G_3_	NM_004526	4171	MCM2 minichromosome maintenance deficient 2, mitotin (S. cerevisiae)
MELK	G_3_	NM_014791	9833	maternal embryonic leucine zipper kinase
MYBL2	G_3_	NM_002466	4605	v-myb myeloblastosis viral oncogene homolog (avian)-like 2
NA	G_3_	BE966236	NA	NA
NCAPG	G_3_	NM_022346	64151	non-SMC condensin I complex, subunit G
POLQ	G_3_	NM_006596	10721	polymerase (DNA directed), theta
PRC1	G_3_	NM_003981	9055	protein regulator of cytokinesis 1
RRM2	G_3_	BC001886	6241	ribonucleotide reductase M2 polypeptide
TIMELESS	G_3_	NM_003920	8914	timeless homolog (Drosophila)
TRIP13	G_3_	NM_004237	9319	thyroid hormone receptor interactor 13
Transcripts whose expression is inversely associated with *POLQ* expression				
CX3CR	G_1_	U20350	1524	chemokine (C-X3-C motif) receptor 1
^s^ G_1_ and G_3_ are the sets of genes with increased expression in histologic grade 1 and 3 tumors, respectively				

### *POLQ* overexpression confers a poor prognosis that is independent of published prognostic signatures

**Fig. 4:**
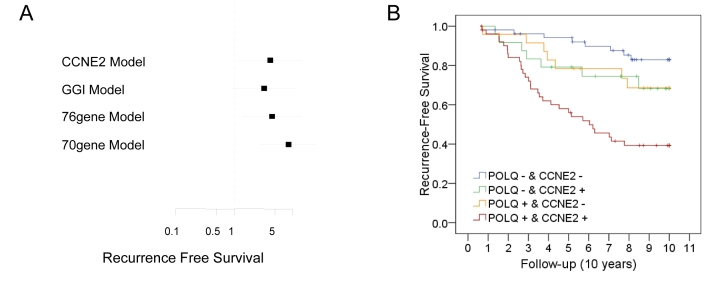
*POLQ* expression shows prognostic potential in multivariate models including clinical variables, published signatures and *CCNE2* A) Forest plot of *POLQ* Hazard Ratio for Recurrence Free Survival in multivariate analysis of Series 1. Dots represent Hazard Ratios (dimensions are proportional to dataset size) and grey bars the 95% confidence intervals. In each analysis, a multivariate model including *POLQ* expression, all significant clinical variables, *CCNE2* expression, and published signature scores (GGI, 76-gene or 70-gene signature) is derived. The expression of *POLQ*, signature scores and *CCNE2* are entered in these models as continuous ranked variables, normalised between 0 (lowest rank) and 1 (highest rank). See methods for more details. B) Kaplan-Meier plots of Series 1 data. *POLQ* and *CCNE2* expression divided by median value (- indicates below median, + above median). A Helmert contrasts analysis demonstrated that tumours overexpressing both *POLQ* and *CCNE2* were associated with worse outcomes than the average of the other groups (HR 3.26; 95% CI 1.88 to 5.66; p<0.001)

As *POLQ* has several genes in common with the GGI signature, and is itself part of the GGI and ‘76 gene’ signatures, we assessed whether *POLQ* expression remained an independent predictor of relapse when these signatures were included in a multivariate analysis of the data from Series 1 (Fig 4A and supplementary table 6). *POLQ* expression remained a strong, independent predictor of disease relapse after statistical consideration of these validated expression profiles and reinforces the close association between *POLQ* expression and adverse outcome.

### The poor prognosis associated with *POLQ* expression is independent of Cyclin E expression

*CCNE2* (cyclin E) is the only gene that is a component of all three expression signatures and which is also co-expressed with *POLQ*. As cyclin E overexpression has been identified as being independently associated with an adverse outcome in breast cancer patients [26], we considered whether the adverse prognosis associated with *POLQ* expression may simply be due to the observation that *CCNE2* is often co-expressed with *POLQ*. We therefore performed a multivariate analysis of the data from Series 1 that included *CCNE2* expression and found that *POLQ* and CCNE2 were both independently associated with an increase in RFS (Fig 4A). It is notable that tumours that overexpress both *POLQ* and *CCNE2* confer an extremely poor prognosis relative to the other groups (HR 3.26; 95% CI 1.88 to 5.66; p<0.001) (Fig 4B). Tumours that do not overexpress either gene are associated with a good prognosis, and those that overexpress only one of the genes are associated with an intermediate prognosis. This data suggests that the biological mechanisms by which *POLQ* and *CCNE2* confer a poor prognosis might be independent of each other. These results could not be confirmed in the other datasets considered, where *POLQ* lost significance after inclusion of *CCNE2*. However it should be noted that Series 1 is the only one in which patients did not receive systemic chemotherapy which is a potential confounding factor for prognostic analysis.

## DISCUSSION

We have recently demonstrated that tumour cells depleted of *POLQ* are rendered more sensitive to radiotherapy and that its limited expression in normal tissues made POLQ a potentially exploitable clinical target [9]. In this study we have demonstrated that *POLQ* is frequently upregulated in breast cancers. Although *POLQ* overexpression has previously been demonstrated in lung, gastric and colorectal cancers [10], to the best of our knowledge, this has not previously been shown in breast cancer.

In this current study we have demonstrated strong associations between *POLQ* expression and the presence of other individual factors such as tumour grade and ER negative disease that are known to confer an adverse prognosis. We have also demonstrated that *POLQ* overexpression is associated with markedly increased rates of disease relapse, and using multivariate analysis, that these increased failure rates are independent of its association with features like tumour grade and ER status.

The mechanisms by which *POLQ* overexpression causes these adverse outcomes are not presently clear. *POLQ* associated radioresistance is likely to contribute to these findings and further work is required to assess whether *POLQ* expression increases the tumour cell resistance to the cytotoxic and endocrine treatments typically used to treat breast cancers. The co-expression of *POLQ* with genes linked to pathways associated with tumour progression, as well as several genes that are contained within the gene expression grade index, suggests that *POLQ* overexpression promotes a more aggressive phenotype, increasing the likelihood of disease recurrence.

The clinical significance of tumour expression of *POLQ* has not previously been examined in detail. A previous study in colorectal cancer correlated the expression levels of genes involved in DNA replication with clinical outcomes in 74 patients with colorectal cancer [27]. Although *POLQ* was not independently associated with adverse outcome, its co-overexpression with at least three other genes involved in DNA replication ‘firing’ (from among *CDC45, CDC6, CDT1, SLD5, MCM2*, and *MCM7*) was associated with a worse overall survival. The overall significance of *POLQ* on this finding is not clear since *MCM7* overexpression was shown to independently be associated with adverse survival rates. This group suggested that the expression of these genes could produce a more aggressive tumour phenotype by contributing to ‘replication stress’. As POLQ is known to repair DNA damage in an error-prone fashion [1, 2], it would seem likely that the poor prognosis that we have described in this study is partially due to POLQ contributing to increased replication stress and genomic instability.

To the best of our knowledge this is the first study to demonstrate an adverse association with *POLQ* expression in patients with breast cancer. In recent years, attempts have been made to identify gene expression signatures that are capable of predicting patient outcomes with greater accuracy than is currently achievable in routine clinical practice. It is possible that specific gene expression profiles could identify the likelihood of response to individual therapies, enabling clinicians to refine the adjuvant therapy offered to individual patients. The GGI signature [25] identified 97 genes with differential expression between low and high grade breast carcinomas. This signature enabled a more accurate and refined assessment of the risk of disease recurrence in patients with intermediate grade disease. Subsequent studies have confirmed the ability of the GGI signature to accurately predict disease relapse [13, 28]. A separate expression profile has been created to more accurately identify patients at risk of developing metastatic disease [16]. This study used tumours derived from patients who did not receive adjuvant systemic therapy, thereby eliminating potentially confounding predictive factors occurring as a result of systemic treatment. The resulting ‘76 gene’ signature was shown to predict both distant failure as well as overall survival. Further studies have reinforced the prognostic accuracy of this gene signature [29, 30]. A third gene expression profile utilising a 70 gene signature has also been shown to predict clinical outcome [24] and has also been subsequently validated [31]. The prognostic effect of *POLQ* expression on its own has not previously been assessed, but it is interesting to note that *POLQ* is a component of both the GGI and the '76 gene' expression profiles. Given the large differences that we have shown in relapse rates on the basis of *POLQ* expression, and that these differences are maintained on multivariate analyses that include these signatures, it is possible that *POLQ* may be amongst the most important determinants within these signatures.

Pathway analysis identified several genes, including Cyclin E, that were frequently co-expressed with *POLQ*. Cyclin E over expression has been identified as being associated with an adverse outcome in breast cancer patients [26]. It is the only gene that is a component of all three gene expression signatures and which is also frequently co-expressed with *POLQ*. Cyclin E binds to cyclin-dependent kinase-2 (cdk-2), permitting the transition from G1 to S-phase [32]. Increased cyclin E induces enhanced cdk-2 activity, accelerating G1/S transition [33]. There is substantial evidence to suggest that *CCNE* overexpression confers a poor prognosis in breast cancer. A recent meta-analysis of 12 independent studies involving 2,534 patients, demonstrated that the combined HR estimate for overall survival and breast cancer specific survival was 2.98 (95% CI, 1.85–4.78) and 2.86 (95% CI, 1.85– 4.41) in univariate and multivariate analysis, respectively [34]. Although there is ongoing debate as to which fragments of cyclin E are important in predicting outcome [35], the evidence supporting its use in routine clinical assessment have led for calls for large scale clinical trials [34]. In this study we have again confirmed that cyclin E overexpression was associated with a poor clinical prognosis on multivariate analysis. In addition we have shown that tumours expressing both *POLQ* and *CCNE2* are associated with an extremely poor outcome. This suggests that these genes confer a poor prognosis through separate mechanisms. Larger studies are required to investigate whether the risk of relapse from tumours overexpressing cyclin E could be better assessed if further stratified by *POLQ* expression levels.

Independently of its association with other known poor pathological features, *POLQ* overexpression is associated with increased relapse rates. This is the first study to demonstrate that *POLQ* overexpression is associated with an extremely poor outcome in breast cancer on both univariate and multivariate analysis.

We believe that the poor prognosis associated with *POLQ* expression, the known radiosensitivity induced by its depletion, and its highly limited normal tissue expression makes POLQ an extremely appealing target for clinical exploitation.

## SUPPLEMENTAL TABLES

Supplementary Table 1

Supplementary Table 2

Supplementary Table 3

Supplementary Table 4

Supplementary Table 5

Supplementary Table 6
