# Supplementary material for: Overexpression of POLQ Confers a Poor Prognosis in Early Breast Cancer Patients
Source: Oncotarget. 2010 Jul 9;1(3):175–84. doi: 10.18632/oncotarget.124 (PMC2917771; doi:10.18632/oncotarget.124)
Supplement: Supplementary Table 1 [file oncotarget-01-175-s001.doc]

**Table S1. Demographics, clinic-pathological characteristics and treatment for Series 1 (N=152) and Series 2 (N=127) analysed in this study**

| ***Series 1*** | | | | | | |
| --- | --- | --- | --- | --- | --- | --- |
| **Continous/Ordinal Covariates** | *Mean* | *Median* | *St.Dev.* | *Minimum* | *Maximum* | *Missing* |
| Age at operation (yrs) | 59.3 | 61 | 11 | 32 | 86 | 0 |
| Tumour size (cm) | 2.5 | 2.1 | 1.3 | 0.2 | 9.0 | 0 |
| Number of nodes involved | 0.8 | 0 | 2.4 | 0 | 16 | 1 |
| **Categorical Covariates** | *Value* | *Frequency* | *Percent* | *Missing* |  |  |
| ER status* | Negative | 26 | 17 | 4 |  |  |
|  | Positive | 122 | 80 |  |  |  |
| Tumor Grade^ζ^ | 1 | 23 | 15 | 23 |  |  |
|  | 2 | 74 | 49 |  |  |  |
|  | 3 | 32 | 21 |  |  |  |
| Histology | Ductal | 122 | 80 | 0 |  |  |
|  | Lobular | 22 | 15 |  |  |  |
|  | Mixed | 5 | 3 |  |  |  |
|  | Others | 3 | 2 |  |  |  |
| **Treatment** |  |  |  |  |  |  |
| Tamoxifen | No | 61 | 40 |  |  |  |
|  | Yes | 91 | 60 |  |  |  |
| Radiotherapy | No | 26 | 17 |  |  |  |
|  | Yes | 126 | 83 |  |  |  |
| Chemotherapy (CMF) | No | 152 | 100 |  |  |  |
|  | Yes | 0 | 0 |  |  |  |
| ***Series 2*** | | | | | | |
| **Continous/Ordinal Covariates** | *Mean* | *Median* | *St.Dev.* | *Minimum* | *Maximum* | *Missing* |
| Age at operation (yrs) | 53.3 | 54 | 10.4 | 26 | 73 | 0 |
| Tumour size (cm) | 2.7 | 2.5 | 1.4 | 0.0 | 7.0 | 0 |
| Number of nodes involved | 2.1 | 1 | 3.1 | 0 | 15 | 1 |
| **Categorical Covariates** | *Value* | *Frequency* | *Percent* | *Missing* |  |  |
| ER status* | Negative | 50 | 39 | 0 |  |  |
|  | Positive | 77 | 61 |  |  |  |
| Tumor Grade^ζ^ | 1 | 28 | 22 | 16 |  |  |
|  | 2 | 48 | 38 |  |  |  |
|  | 3 | 35 | 28 |  |  |  |
| Histology | Ductal | 92 | 72 | 0 |  |  |
|  | Lobular | 15 | 12 |  |  |  |
|  | Mixed | 13 | 10 |  |  |  |
|  | Others | 7 | 6 |  |  |  |
| **Treatment** |  |  |  |  |  |  |
| Tamoxifen | No | 51 | 40 |  |  |  |
|  | Yes | 76 | 60 |  |  |  |
| Radiotherapy | No | 21 | 17 |  |  |  |
|  | Yes | 106 | 83 |  |  |  |
| Chemotherapy (CMF) | No | 74 | 58 |  |  |  |
|  | Yes | 53 | 42 |  |  |  |

* Method by EORTC Breast Cancer Co-operative group [1]; tumours with cytoplasm ER levels ≥ 10 fmol/mg of protein were considered positive. For 11 tumours where this measurement was missing we used gene expression threshold instead as previously described [2].

^ζ^ Modified Bloom and Richardson method [3].

**References**

1. Group EOfRaToCBCC (1980) Revision of the standards for the assessment of hormone receptors in human breast cancer; report of the second E.O.R.T.C. Workshop, held on 16-17 March, 1979, in the Netherlands Cancer Institute. *Eur J Cancer* 16:1513-1515.

2. Gong Y, et al. (2007) Determination of oestrogen-receptor status and ERBB2 status of breast carcinoma: a gene-expression profiling study. *Lancet Oncol* 8:203-211.

3. Elston C (1987) in *Grading of invasive carcinoma of the breast*, eds. Page DAnderson T (Churchill Livingstone, Edinburgh), pp. 300-311.
