# Supplementary material for: Overexpression of POLQ Confers a Poor Prognosis in Early Breast Cancer Patients
Source: Oncotarget. 2010 Jul 9;1(3):175–84. doi: 10.18632/oncotarget.124 (PMC2917771; doi:10.18632/oncotarget.124)
Supplement: Supplementary Table 2 [file oncotarget-01-175-s002.doc]

Supplementary Table 2. Two probesets matched to *POLQ* gene ID 10721 on the Affymetrix U133A gene array; one on Illumina Human RefSeq-8 v1 arrays.

| Probe Set ID | Gene Symbol | Transcript ID | RefSeq Protein ID | Target Sequence |
| --- | --- | --- | --- | --- |
| 219510_at | POLQ | AF052573 /// AY032677 /// AY338826 /// ENST00000264233 /// ENST00000393672 /// NM_199420 /// uc003eed.1 /// uc003eee.1 | NP_955452 | tagcactttggttccacatctgtctgggtaaaccatgaagaaaatgaagctgctgcctcaatcgacccagacagcagccataggcagataaagatttggtttcaccctggtggtggtaggcatcgtgtgtgactttttttcctctaatatcaattttacagtacggaaatagtattttaaaatagtattggctaataaattatgaattctataaagtagtaagacttggtatggttggagtgtaggaatgaatattcatgaaatgtttcttattgcttttccttccctaattcatacaatgaatgtatttggaatacttacatattataaaataaactatacctcttcaagaggtatcctgttctgtaagatcagatgtttttattgcaggtcaatataatactgccagagacagaaaatacccccttatcagtcccttagtgcctctttctgtttgtggcatggtgagaaaacccatgctgaaaagattgtactttgtgatcccaatcaga |
| 207746_at | POLQ | AF090919 | NP_955452 | aaagcaggtttcaccggtcatctatcatggccttaaaaataagtcatttattatacagtggctaagattgtgggctctaggatcagactgccttagttcaacttttggcctcatcacttagtaatcatataaccttgggtaagtatttaacctctcttacgattccatttcctcatttgtaaaatggagataataatacccacctcagggctgatagtctttgatgaatgtttggtatctgaataaatgtttaataacatctattatttctagtaaattctcccataaacattatgtaagtcatttgccaaattacctaactactcctactcctgttccctctctaaaacgtgaagactgttggcagtgttagtatgctgaatgcttgtggttagtggtcttgtatacttctctcaccattcgagttgtatgcccttcaagagttagttgtgttcccaaacttgttcatgtcagtt |
| ILMN_ 6520504 | POLQ | NM_006596 | NP_955452 | taatactgccagagacagaaaatacccccttatcagtcccttagtgcctc |
