# Supplementary material for: Overexpression of POLQ Confers a Poor Prognosis in Early Breast Cancer Patients
Source: Oncotarget. 2010 Jul 9;1(3):175–84. doi: 10.18632/oncotarget.124 (PMC2917771; doi:10.18632/oncotarget.124)
Supplement: Supplementary Table 3 [file oncotarget-01-175-s003.doc]

**Table S3.**Datasets used for the seed-clustering data-mining

| **Name** | **Size** | **Site** | **Reference** |
| --- | --- | --- | --- |
| GSE6532Oxf | 149 | Breast | [1] |
| GSE6532KI | 178 | Breast | [1] |
| GSE6532GUY | 87 | Breast | [1] |
| GSE2034 | 286 | Breast | [2] |
| GSE3494 | 315 | Breast | [3] |

**References**

1. Loi S, Haibe-Kains B, Desmedt C, Wirapati P, Lallemand F, Tutt AM, Gillet C, Ellis P, Ryder K, Reid JF, Daidone MG, Pierotti MA, Berns EM, Jansen MP, Foekens JA, Delorenzi M, Bontempi G, Piccart MJ, Sotiriou C (2008) Predicting prognosis using molecular profiling in estrogen receptor-positive breast cancer treated with tamoxifen. *BMC Genomics* **9:** 239

2. Carroll JS, Meyer CA, Song J, Li W, Geistlinger TR, Eeckhoute J, Brodsky AS, Keeton EK, Fertuck KC, Hall GF, Wang Q, Bekiranov S, Sementchenko V, Fox EA, Silver PA, Gingeras TR, Liu XS, Brown M (2006) Genome-wide analysis of estrogen receptor binding sites. *Nat Genet* **38:** 1289-97

3. Miller LD, Smeds J, George J, Vega VB, Vergara L, Ploner A, Pawitan Y, Hall P, Klaar S, Liu ET, Bergh J (2005) An expression signature for p53 status in human breast cancer predicts mutation status, transcriptional effects, and patient survival. *Proc Natl Acad Sci U S A* **102:** 13550-5
