# Supplementary material for: Overexpression of POLQ Confers a Poor Prognosis in Early Breast Cancer Patients
Source: Oncotarget. 2010 Jul 9;1(3):175–84. doi: 10.18632/oncotarget.124 (PMC2917771; doi:10.18632/oncotarget.124)
Supplement: Supplementary Table 4 [file oncotarget-01-175-s004.doc]

Supplementary Table 4. Results of the multivariate analyses performed on each data series. Data summarised in Fig 2C.

**Series 1: 152 breast cancer cases on Affymetrix arrays**

Multivariate analysis:

|  |  |  |  |  | 95.0% CI for HR | |
| --- | --- | --- | --- | --- | --- | --- |
|  | B | SE | p | Hazard Ratio | Lower | Upper |
| POLQ2 score | 2.090 | 0.633 | 0.001 | 8.086 | 2.340 | 27.948 |
| Age | 0.008 | 0.017 | 0.625 | 1.009 | 0.975 | 1.043 |
| Grade | 0.028 | 0.266 | 0.916 | 0.972 | 0.577 | 1.638 |
| Tumour Size | 0.243 | 0.103 | 0.018 | 1.275 | 1.042 | 1.561 |
| ER status | -0.622 | 0.580 | 0.284 | 0.537 | 0.172 | 1.673 |
| Tamoxifen | 0.218 | 0.505 | 0.666 | 1.244 | 0.462 | 3.349 |
| Nodal status | -0.029 | 0.077 | 0.711 | 0.972 | 0.836 | 1.130 |

Backward Likelihood Reduced model :

|  |  |  |  |  | 95.0% CI for HR | |
| --- | --- | --- | --- | --- | --- | --- |
|  | B | SE | p | Hazard Ratio | Lower | Upper |
| Tumour Size | 0.227 | 0.085 | 0.007 | 1.255 | 1.063 | 1.482 |
| POLQ2 score | 2.132 | 0.576 | 0.000 | 8.435 | 2.727 | 26.089 |

**Series 2**: 127 breast cancer cases on Illumina arrays

| Multivariate analysis: |  |  |  |  |  |  |
| --- | --- | --- | --- | --- | --- | --- |
|  | |  |  |  |  |  |
|  |  |  |  |  | 95.0% CI for Haxard Ratio |  |
| Variables | B | SE | Sig. | Hazard Ratio | Lower | Upper |
| Age (Decade) | 0.487 | 0.171 | 0.004 | 1.628 | 1.165 | 2.275 |
| Tumor Size (cm) | 0.129 | 0.118 | 0.274 | 1.138 | 0.903 | 1.435 |
| Nodes Involved | 0.266 | 0.045 | 0.000 | 1.304 | 1.194 | 1.426 |
| ER status | -0.236 | 0.359 | 0.510 | 0.790 | 0.391 | 1.596 |
| Tamoxifen | -0.145 | 0.358 | 0.686 | 0.865 | 0.429 | 1.747 |
| Grade | 0.122 | 0.289 | 0.672 | 1.130 | 0.642 | 1.990 |
| POLQ | 1.627 | 0.700 | 0.020 | 5.087 | 1.290 | 20.064 |
|  |  |  |  |  |  |  |
|  |  |  |  |  |  |  |
| Backward Likelihood Reduced model : | |  |  |  |  |  |
| Age (Decade) | 0.454 | 0.163 | 0.005 | 1.574 | 1.143 | 2.169 |
| Nodes Involved | 0.268 | 0.043 | 0.000 | 1.307 | 1.202 | 1.423 |
| POLQ | 1.939 | 0.611 | 0.002 | 6.952 | 2.099 | 23.028 |

**GSE2034 dataset: 286 breast cancer cases on Affymetrix U133A arrays**

Relapse-Free Survival

Multivariate analysis:

|  |  |  |  |  | 95.0% CI for HR | |
| --- | --- | --- | --- | --- | --- | --- |
|  | B | SE | P | Hazard Ratio | Lower | Upper |
| POLQ2 score | 0.737 | 0.355 | 0.038 | 2.090 | 1.042 | 4.192 |
| ER Status | 0.113 | 0.229 | 0.622 | 1.119 | 0.715 | 1.754 |

Backward Likelihood Reduced model:

|  |  |  |  |  | 95.0% CI for HR | |
| --- | --- | --- | --- | --- | --- | --- |
|  | B | SE | P | Hazard Ratio | Lower | Upper |
| POLQ2 score | 0.6949 | 0.3451 | 0.0441 | 2.0034 | 1.0186 | 3.9402 |

**GSE3494 dataset: 251 breast cancer cases on Affymetrix U133A arrays**

Disease specific survival (Gene expression as continuous variable : samples are ranked from low to high expression, and the ranks are normalised between 0 and 1)

|  |  |  |  |  | 95.0% CI for HR | |
| --- | --- | --- | --- | --- | --- | --- |
|  | B | SE | p | Hazard Ratio | Lower | Upper |
| POLQ2 Score | 0.806 | 0.478 | 0.092 | 2.239 | 0.877 | 5.712 |
