# Supplementary material for: Overexpression of POLQ Confers a Poor Prognosis in Early Breast Cancer Patients
Source: Oncotarget. 2010 Jul 9;1(3):175–84. doi: 10.18632/oncotarget.124 (PMC2917771; doi:10.18632/oncotarget.124)
Supplement: Supplementary Table 5 [file oncotarget-01-175-s005.doc]

Supplementary Table 5. Seed-clustering with bootstrap resampling identified 97 genes co-expressed with *POLQ.* Probes with a Connectivity Normalised Rank greater than 0.5 are shown; this corresponds to probes that are significantly co-expressed with both *POLQ* seeds in at least 2 datasets.

| Gene Symbol | RefSeq | Unigene | Name | Connectivity Normalised Rank (0=lowest, 1=highest) |
| --- | --- | --- | --- | --- |
| PRB3 | [NM_006249](http://www.ncbi.nlm.nih.gov/entrez/query.fcgi?cmd=search&db=nucleotide&term=NM_006249) | [Hs.73031](http://www.ncbi.nlm.nih.gov/entrez/query.fcgi?cmd=search&db=unigene&term=Hs.73031&dopt=unigene) | proline-rich protein BstNI subfamily 3 | 0.995104 |
| LLGL1 | [D50550](http://www.ncbi.nlm.nih.gov/entrez/query.fcgi?cmd=search&db=nucleotide&term=D50550) | [Hs.513983](http://www.ncbi.nlm.nih.gov/entrez/query.fcgi?cmd=search&db=unigene&term=Hs.513983&dopt=unigene) | lethal giant larvae homolog 1 (Drosophila) | 0.991976 |
| GSK3A | [NM_019884](http://www.ncbi.nlm.nih.gov/entrez/query.fcgi?cmd=search&db=nucleotide&term=NM_019884) | [Hs.466828](http://www.ncbi.nlm.nih.gov/entrez/query.fcgi?cmd=search&db=unigene&term=Hs.466828&dopt=unigene) | glycogen synthase kinase 3 alpha | 0.991949 |
| ESPL1 | [D79987](http://www.ncbi.nlm.nih.gov/entrez/query.fcgi?cmd=search&db=nucleotide&term=D79987) | [Hs.153479](http://www.ncbi.nlm.nih.gov/entrez/query.fcgi?cmd=search&db=unigene&term=Hs.153479&dopt=unigene) | extra spindle poles like 1 (S. cerevisiae) | 0.988352 |
| POLA2 | [NM_002689](http://www.ncbi.nlm.nih.gov/entrez/query.fcgi?cmd=search&db=nucleotide&term=NM_002689) | [Hs.201897](http://www.ncbi.nlm.nih.gov/entrez/query.fcgi?cmd=search&db=unigene&term=Hs.201897&dopt=unigene) | polymerase (DNA directed), alpha 2 (70kD subunit) | 0.986047 |
| MINK1 | [AB041926](http://www.ncbi.nlm.nih.gov/entrez/query.fcgi?cmd=search&db=nucleotide&term=AB041926) | [Hs.443417](http://www.ncbi.nlm.nih.gov/entrez/query.fcgi?cmd=search&db=unigene&term=Hs.443417&dopt=unigene) | misshapen-like kinase 1 (zebrafish) | 0.984246 |
| MTRF1L | [AU145135](http://www.ncbi.nlm.nih.gov/entrez/query.fcgi?cmd=search&db=nucleotide&term=AU145135) | [Hs.225836](http://www.ncbi.nlm.nih.gov/entrez/query.fcgi?cmd=search&db=unigene&term=Hs.225836&dopt=unigene) | mitochondrial translational release factor 1-like | 0.969734 |
| FLJ10719 | [W74442](http://www.ncbi.nlm.nih.gov/entrez/query.fcgi?cmd=search&db=nucleotide&term=W74442) | [Hs.513126](http://www.ncbi.nlm.nih.gov/entrez/query.fcgi?cmd=search&db=unigene&term=Hs.513126&dopt=unigene) | NA | 0.968868 |
| MYBL2 | [NM_002466](http://www.ncbi.nlm.nih.gov/entrez/query.fcgi?cmd=search&db=nucleotide&term=NM_002466) | [Hs.179718](http://www.ncbi.nlm.nih.gov/entrez/query.fcgi?cmd=search&db=unigene&term=Hs.179718&dopt=unigene) | v-myb myeloblastosis viral oncogene homolog (avian)-like 2 | 0.968597 |
| BYSL | [NM_004053](http://www.ncbi.nlm.nih.gov/entrez/query.fcgi?cmd=search&db=nucleotide&term=NM_004053) | [Hs.106880](http://www.ncbi.nlm.nih.gov/entrez/query.fcgi?cmd=search&db=unigene&term=Hs.106880&dopt=unigene) | bystin-like | 0.963122 |
| hCAP-H2 | [BC001298](http://www.ncbi.nlm.nih.gov/entrez/query.fcgi?cmd=search&db=nucleotide&term=BC001298) | [Hs.180903](http://www.ncbi.nlm.nih.gov/entrez/query.fcgi?cmd=search&db=unigene&term=Hs.180903&dopt=unigene) | NA | 0.960984 |
| BMP7 | [BC004248](http://www.ncbi.nlm.nih.gov/entrez/query.fcgi?cmd=search&db=nucleotide&term=BC004248) | [Hs.473163](http://www.ncbi.nlm.nih.gov/entrez/query.fcgi?cmd=search&db=unigene&term=Hs.473163&dopt=unigene) | bone morphogenetic protein 7 (osteogenic protein 1) | 0.959162 |
| MYST3 | [NM_006766](http://www.ncbi.nlm.nih.gov/entrez/query.fcgi?cmd=search&db=nucleotide&term=NM_006766) | [Hs.491577](http://www.ncbi.nlm.nih.gov/entrez/query.fcgi?cmd=search&db=unigene&term=Hs.491577&dopt=unigene) | MYST histone acetyltransferase (monocytic leukemia) 3 | 0.958289 |
| PPP2R5B | [NM_006244](http://www.ncbi.nlm.nih.gov/entrez/query.fcgi?cmd=search&db=nucleotide&term=NM_006244) | [Hs.75199](http://www.ncbi.nlm.nih.gov/entrez/query.fcgi?cmd=search&db=unigene&term=Hs.75199&dopt=unigene) | protein phosphatase 2, regulatory subunit B (B56), beta isoform | 0.946218 |
| ZFX | [R51161](http://www.ncbi.nlm.nih.gov/entrez/query.fcgi?cmd=search&db=nucleotide&term=R51161) | [Hs.370424](http://www.ncbi.nlm.nih.gov/entrez/query.fcgi?cmd=search&db=unigene&term=Hs.370424&dopt=unigene) | zinc finger protein, X-linked | 0.941965 |
| SIL | [NM_003035](http://www.ncbi.nlm.nih.gov/entrez/query.fcgi?cmd=search&db=nucleotide&term=NM_003035) | [Hs.525198](http://www.ncbi.nlm.nih.gov/entrez/query.fcgi?cmd=search&db=unigene&term=Hs.525198&dopt=unigene) | TAL1 (SCL) interrupting locus | 0.940061 |
| DDX11 | [NM_030653](http://www.ncbi.nlm.nih.gov/entrez/query.fcgi?cmd=search&db=nucleotide&term=NM_030653) | [Hs.443960](http://www.ncbi.nlm.nih.gov/entrez/query.fcgi?cmd=search&db=unigene&term=Hs.443960&dopt=unigene) | DEAD/H (Asp-Glu-Ala-Asp/His) box polypeptide 11 (CHL1-like helicase homolog, S. cerevisiae) | 0.936715 |
| TOP2A | [AU159942](http://www.ncbi.nlm.nih.gov/entrez/query.fcgi?cmd=search&db=nucleotide&term=AU159942) | [Hs.156346](http://www.ncbi.nlm.nih.gov/entrez/query.fcgi?cmd=search&db=unigene&term=Hs.156346&dopt=unigene) | topoisomerase (DNA) II alpha 170kDa | 0.935946 |
| GTSE1 | [BC006325](http://www.ncbi.nlm.nih.gov/entrez/query.fcgi?cmd=search&db=nucleotide&term=BC006325) | [Hs.386189](http://www.ncbi.nlm.nih.gov/entrez/query.fcgi?cmd=search&db=unigene&term=Hs.386189&dopt=unigene) | G-2 and S-phase expressed 1 | 0.93441 |
| HCAP-G | [NM_022346](http://www.ncbi.nlm.nih.gov/entrez/query.fcgi?cmd=search&db=nucleotide&term=NM_022346) | [Hs.446201](http://www.ncbi.nlm.nih.gov/entrez/query.fcgi?cmd=search&db=unigene&term=Hs.446201&dopt=unigene) | NA | 0.929025 |
| D21S2056E | [NM_003683](http://www.ncbi.nlm.nih.gov/entrez/query.fcgi?cmd=search&db=nucleotide&term=NM_003683) | [Hs.110757](http://www.ncbi.nlm.nih.gov/entrez/query.fcgi?cmd=search&db=unigene&term=Hs.110757&dopt=unigene) | NA | 0.927897 |
| NPAL2 | [NM_024759](http://www.ncbi.nlm.nih.gov/entrez/query.fcgi?cmd=search&db=nucleotide&term=NM_024759) | [Hs.309489](http://www.ncbi.nlm.nih.gov/entrez/query.fcgi?cmd=search&db=unigene&term=Hs.309489&dopt=unigene) | NIPA-like domain containing 2 | 0.92778 |
| STK6 | [NM_003158](http://www.ncbi.nlm.nih.gov/entrez/query.fcgi?cmd=search&db=nucleotide&term=NM_003158) | [Hs.250822](http://www.ncbi.nlm.nih.gov/entrez/query.fcgi?cmd=search&db=unigene&term=Hs.250822&dopt=unigene) | serine/threonine kinase 6 | 0.924998 |
| MRPL2 | [NM_015950](http://www.ncbi.nlm.nih.gov/entrez/query.fcgi?cmd=search&db=nucleotide&term=NM_015950) | [Hs.55041](http://www.ncbi.nlm.nih.gov/entrez/query.fcgi?cmd=search&db=unigene&term=Hs.55041&dopt=unigene) | mitochondrial ribosomal protein L2 | 0.914122 |
| KRT8L2 | [AI357616](http://www.ncbi.nlm.nih.gov/entrez/query.fcgi?cmd=search&db=nucleotide&term=AI357616) | [Hs.101651](http://www.ncbi.nlm.nih.gov/entrez/query.fcgi?cmd=search&db=unigene&term=Hs.101651&dopt=unigene) | keratin 8-like 2 | 0.905579 |
| ZBTB7A | [AF097916](http://www.ncbi.nlm.nih.gov/entrez/query.fcgi?cmd=search&db=nucleotide&term=AF097916) | [Hs.465623](http://www.ncbi.nlm.nih.gov/entrez/query.fcgi?cmd=search&db=unigene&term=Hs.465623&dopt=unigene) | zinc finger and BTB domain containing 7A | 0.896152 |
| METAP2 | [NM_006838](http://www.ncbi.nlm.nih.gov/entrez/query.fcgi?cmd=search&db=nucleotide&term=NM_006838) | [Hs.444986](http://www.ncbi.nlm.nih.gov/entrez/query.fcgi?cmd=search&db=unigene&term=Hs.444986&dopt=unigene) | methionyl aminopeptidase 2 | 0.887015 |
| LOC155060 | [AU151157](http://www.ncbi.nlm.nih.gov/entrez/query.fcgi?cmd=search&db=nucleotide&term=AU151157) | [Hs.490512](http://www.ncbi.nlm.nih.gov/entrez/query.fcgi?cmd=search&db=unigene&term=Hs.490512&dopt=unigene) | NA | 0.882748 |
| LIME1 | [NM_017806](http://www.ncbi.nlm.nih.gov/entrez/query.fcgi?cmd=search&db=nucleotide&term=NM_017806) | [Hs.233220](http://www.ncbi.nlm.nih.gov/entrez/query.fcgi?cmd=search&db=unigene&term=Hs.233220&dopt=unigene) | Lck interacting transmembrane adaptor 1 | 0.863774 |
| GFER | [NM_005262](http://www.ncbi.nlm.nih.gov/entrez/query.fcgi?cmd=search&db=nucleotide&term=NM_005262) | [Hs.27184](http://www.ncbi.nlm.nih.gov/entrez/query.fcgi?cmd=search&db=unigene&term=Hs.27184&dopt=unigene) | growth factor, augmenter of liver regeneration (ERV1 homolog, S. cerevisiae) | 0.861185 |
| RBM21 | [NM_022830](http://www.ncbi.nlm.nih.gov/entrez/query.fcgi?cmd=search&db=nucleotide&term=NM_022830) | [Hs.256184](http://www.ncbi.nlm.nih.gov/entrez/query.fcgi?cmd=search&db=unigene&term=Hs.256184&dopt=unigene) | RNA binding motif protein 21 | 0.849952 |
| DNAJC4 | [NM_005528](http://www.ncbi.nlm.nih.gov/entrez/query.fcgi?cmd=search&db=nucleotide&term=NM_005528) | [Hs.172847](http://www.ncbi.nlm.nih.gov/entrez/query.fcgi?cmd=search&db=unigene&term=Hs.172847&dopt=unigene) | DnaJ (Hsp40) homolog, subfamily C, member 4 | 0.84681 |
| LAT2 | [BC006080](http://www.ncbi.nlm.nih.gov/entrez/query.fcgi?cmd=search&db=nucleotide&term=BC006080) | [Hs.56607](http://www.ncbi.nlm.nih.gov/entrez/query.fcgi?cmd=search&db=unigene&term=Hs.56607&dopt=unigene) | linker for activation of T cells family, member 2 | 0.846083 |
| KIAA0101 | [NM_014736](http://www.ncbi.nlm.nih.gov/entrez/query.fcgi?cmd=search&db=nucleotide&term=NM_014736) | [Hs.81892](http://www.ncbi.nlm.nih.gov/entrez/query.fcgi?cmd=search&db=unigene&term=Hs.81892&dopt=unigene) | KIAA0101 | 0.84319 |
| C20orf172 | [NM_024918](http://www.ncbi.nlm.nih.gov/entrez/query.fcgi?cmd=search&db=nucleotide&term=NM_024918) | [Hs.266273](http://www.ncbi.nlm.nih.gov/entrez/query.fcgi?cmd=search&db=unigene&term=Hs.266273&dopt=unigene) | chromosome 20 open reading frame 172 | 0.839673 |
| ST3GAL4 | [NM_006278](http://www.ncbi.nlm.nih.gov/entrez/query.fcgi?cmd=search&db=nucleotide&term=NM_006278) | [Hs.504251](http://www.ncbi.nlm.nih.gov/entrez/query.fcgi?cmd=search&db=unigene&term=Hs.504251&dopt=unigene) | ST3 beta-galactoside alpha-2,3-sialyltransferase 4 | 0.830919 |
| ZNHIT4 | [NM_031288](http://www.ncbi.nlm.nih.gov/entrez/query.fcgi?cmd=search&db=nucleotide&term=NM_031288) | [Hs.410786](http://www.ncbi.nlm.nih.gov/entrez/query.fcgi?cmd=search&db=unigene&term=Hs.410786&dopt=unigene) | zinc finger, HIT type 4 | 0.826217 |
| PLEKHG5 | [AI275938](http://www.ncbi.nlm.nih.gov/entrez/query.fcgi?cmd=search&db=nucleotide&term=AI275938) | [Hs.284232](http://www.ncbi.nlm.nih.gov/entrez/query.fcgi?cmd=search&db=unigene&term=Hs.284232&dopt=unigene) | pleckstrin homology domain containing, family G (with RhoGef domain) member 5 | 0.818154 |
| PELP1 | [BC002875](http://www.ncbi.nlm.nih.gov/entrez/query.fcgi?cmd=search&db=nucleotide&term=BC002875) | [Hs.513883](http://www.ncbi.nlm.nih.gov/entrez/query.fcgi?cmd=search&db=unigene&term=Hs.513883&dopt=unigene) | NA | 0.81699 |
| HLRC1 | [NM_031304](http://www.ncbi.nlm.nih.gov/entrez/query.fcgi?cmd=search&db=nucleotide&term=NM_031304) | [Hs.515064](http://www.ncbi.nlm.nih.gov/entrez/query.fcgi?cmd=search&db=unigene&term=Hs.515064&dopt=unigene) | HEAT-like (PBS lyase) repeat containing 1 | 0.806763 |
| TIMELESS | [NM_003920](http://www.ncbi.nlm.nih.gov/entrez/query.fcgi?cmd=search&db=nucleotide&term=NM_003920) | [Hs.118631](http://www.ncbi.nlm.nih.gov/entrez/query.fcgi?cmd=search&db=unigene&term=Hs.118631&dopt=unigene) | timeless homolog (Drosophila) | 0.793701 |
| MYH14 | [NM_024729](http://www.ncbi.nlm.nih.gov/entrez/query.fcgi?cmd=search&db=nucleotide&term=NM_024729) | [Hs.467142](http://www.ncbi.nlm.nih.gov/entrez/query.fcgi?cmd=search&db=unigene&term=Hs.467142&dopt=unigene) | myosin, heavy polypeptide 14 | 0.792221 |
| FAM3A | [BC002934](http://www.ncbi.nlm.nih.gov/entrez/query.fcgi?cmd=search&db=nucleotide&term=BC002934) | [Hs.289108](http://www.ncbi.nlm.nih.gov/entrez/query.fcgi?cmd=search&db=unigene&term=Hs.289108&dopt=unigene) | family with sequence similarity 3, member A | 0.78676 |
| LOC127406 | [AL353681](http://www.ncbi.nlm.nih.gov/entrez/query.fcgi?cmd=search&db=nucleotide&term=AL353681) | [Hs.112622](http://www.ncbi.nlm.nih.gov/entrez/query.fcgi?cmd=search&db=unigene&term=Hs.112622&dopt=unigene) | NA | 0.779367 |
| GIPC1 | [NM_005716](http://www.ncbi.nlm.nih.gov/entrez/query.fcgi?cmd=search&db=nucleotide&term=NM_005716) | [Hs.6454](http://www.ncbi.nlm.nih.gov/entrez/query.fcgi?cmd=search&db=unigene&term=Hs.6454&dopt=unigene) | GIPC PDZ domain containing family, member 1 | 0.76827 |
| LRP6 | [NM_002336](http://www.ncbi.nlm.nih.gov/entrez/query.fcgi?cmd=search&db=nucleotide&term=NM_002336) | [Hs.210343](http://www.ncbi.nlm.nih.gov/entrez/query.fcgi?cmd=search&db=unigene&term=Hs.210343&dopt=unigene) | low density lipoprotein receptor-related protein 6 | 0.765471 |
| SEC22L2 | [NM_012430](http://www.ncbi.nlm.nih.gov/entrez/query.fcgi?cmd=search&db=nucleotide&term=NM_012430) | [Hs.477361](http://www.ncbi.nlm.nih.gov/entrez/query.fcgi?cmd=search&db=unigene&term=Hs.477361&dopt=unigene) | SEC22 vesicle trafficking protein-like 2 (S. cerevisiae) | 0.741914 |
| ARL6IP2 | [AW301806](http://www.ncbi.nlm.nih.gov/entrez/query.fcgi?cmd=search&db=nucleotide&term=AW301806) | [Hs.190440](http://www.ncbi.nlm.nih.gov/entrez/query.fcgi?cmd=search&db=unigene&term=Hs.190440&dopt=unigene) | ADP-ribosylation factor-like 6 interacting protein 2 | 0.722053 |
| KIFC1 | [BC000712](http://www.ncbi.nlm.nih.gov/entrez/query.fcgi?cmd=search&db=nucleotide&term=BC000712) | [Hs.436912](http://www.ncbi.nlm.nih.gov/entrez/query.fcgi?cmd=search&db=unigene&term=Hs.436912&dopt=unigene) | kinesin family member C1 | 0.71913 |
| PLEKHM2 | [AB020649](http://www.ncbi.nlm.nih.gov/entrez/query.fcgi?cmd=search&db=nucleotide&term=AB020649) | [Hs.145049](http://www.ncbi.nlm.nih.gov/entrez/query.fcgi?cmd=search&db=unigene&term=Hs.145049&dopt=unigene) | pleckstrin homology domain containing, family M (with RUN domain) member 2 | 0.715511 |
| KIAA1217 | [AK022045](http://www.ncbi.nlm.nih.gov/entrez/query.fcgi?cmd=search&db=nucleotide&term=AK022045) | [Hs.445885](http://www.ncbi.nlm.nih.gov/entrez/query.fcgi?cmd=search&db=unigene&term=Hs.445885&dopt=unigene) | KIAA1217 | 0.711276 |
| MXD3 | [NM_031300](http://www.ncbi.nlm.nih.gov/entrez/query.fcgi?cmd=search&db=nucleotide&term=NM_031300) | [Hs.442993](http://www.ncbi.nlm.nih.gov/entrez/query.fcgi?cmd=search&db=unigene&term=Hs.442993&dopt=unigene) | MAX dimerization protein 3 | 0.701903 |
| C20orf67 | [AL162458](http://www.ncbi.nlm.nih.gov/entrez/query.fcgi?cmd=search&db=nucleotide&term=AL162458) | [Hs.472856](http://www.ncbi.nlm.nih.gov/entrez/query.fcgi?cmd=search&db=unigene&term=Hs.472856&dopt=unigene) | chromosome 20 open reading frame 67 | 0.69726 |
| KIAA0894 | [NM_014896](http://www.ncbi.nlm.nih.gov/entrez/query.fcgi?cmd=search&db=nucleotide&term=NM_014896) | [Hs.38621](http://www.ncbi.nlm.nih.gov/entrez/query.fcgi?cmd=search&db=unigene&term=Hs.38621&dopt=unigene) | NA | 0.691729 |
| C14orf172 | [AI679213](http://www.ncbi.nlm.nih.gov/entrez/query.fcgi?cmd=search&db=nucleotide&term=AI679213) | [Hs.525610](http://www.ncbi.nlm.nih.gov/entrez/query.fcgi?cmd=search&db=unigene&term=Hs.525610&dopt=unigene) | chromosome 14 open reading frame 172 | 0.673026 |
| PHF20L1 | [AK022280](http://www.ncbi.nlm.nih.gov/entrez/query.fcgi?cmd=search&db=nucleotide&term=AK022280) | [Hs.304362](http://www.ncbi.nlm.nih.gov/entrez/query.fcgi?cmd=search&db=unigene&term=Hs.304362&dopt=unigene) | PHD finger protein 20-like 1 | 0.670319 |
| GRK6 | [NM_002082](http://www.ncbi.nlm.nih.gov/entrez/query.fcgi?cmd=search&db=nucleotide&term=NM_002082) | [Hs.235116](http://www.ncbi.nlm.nih.gov/entrez/query.fcgi?cmd=search&db=unigene&term=Hs.235116&dopt=unigene) | G protein-coupled receptor kinase 6 | 0.652819 |
| ALDH3B1 | [BC002553](http://www.ncbi.nlm.nih.gov/entrez/query.fcgi?cmd=search&db=nucleotide&term=BC002553) | [Hs.523841](http://www.ncbi.nlm.nih.gov/entrez/query.fcgi?cmd=search&db=unigene&term=Hs.523841&dopt=unigene) | aldehyde dehydrogenase 3 family, member B1 | 0.648404 |
| SUV39H1 | [NM_003173](http://www.ncbi.nlm.nih.gov/entrez/query.fcgi?cmd=search&db=nucleotide&term=NM_003173) | [Hs.522639](http://www.ncbi.nlm.nih.gov/entrez/query.fcgi?cmd=search&db=unigene&term=Hs.522639&dopt=unigene) | suppressor of variegation 3-9 homolog 1 (Drosophila) | 0.646578 |
| POLH | [NM_006502](http://www.ncbi.nlm.nih.gov/entrez/query.fcgi?cmd=search&db=nucleotide&term=NM_006502) | [Hs.439153](http://www.ncbi.nlm.nih.gov/entrez/query.fcgi?cmd=search&db=unigene&term=Hs.439153&dopt=unigene) | polymerase (DNA directed), eta | 0.645134 |
| BRCA1 | [NM_007295](http://www.ncbi.nlm.nih.gov/entrez/query.fcgi?cmd=search&db=nucleotide&term=NM_007295) | [Hs.194143](http://www.ncbi.nlm.nih.gov/entrez/query.fcgi?cmd=search&db=unigene&term=Hs.194143&dopt=unigene) | breast cancer 1, early onset | 0.639475 |
| TSC22D4 | [NM_030935](http://www.ncbi.nlm.nih.gov/entrez/query.fcgi?cmd=search&db=nucleotide&term=NM_030935) | [Hs.469798](http://www.ncbi.nlm.nih.gov/entrez/query.fcgi?cmd=search&db=unigene&term=Hs.469798&dopt=unigene) | TSC22 domain family, member 4 | 0.633456 |
| CDKN3 | [AF213033](http://www.ncbi.nlm.nih.gov/entrez/query.fcgi?cmd=search&db=nucleotide&term=AF213033) | [Hs.84113](http://www.ncbi.nlm.nih.gov/entrez/query.fcgi?cmd=search&db=unigene&term=Hs.84113&dopt=unigene) | cyclin-dependent kinase inhibitor 3 (CDK2-associated dual specificity phosphatase) | 0.625792 |
| DC12 | [AI937543](http://www.ncbi.nlm.nih.gov/entrez/query.fcgi?cmd=search&db=nucleotide&term=AI937543) | [Hs.458320](http://www.ncbi.nlm.nih.gov/entrez/query.fcgi?cmd=search&db=unigene&term=Hs.458320&dopt=unigene) | NA | 0.625254 |
| WBSCR20B | [AI768378](http://www.ncbi.nlm.nih.gov/entrez/query.fcgi?cmd=search&db=nucleotide&term=AI768378) | [Hs.549260](http://www.ncbi.nlm.nih.gov/entrez/query.fcgi?cmd=search&db=unigene&term=Hs.549260&dopt=unigene) | NA | 0.619754 |
| P2RY6 | [NM_004154](http://www.ncbi.nlm.nih.gov/entrez/query.fcgi?cmd=search&db=nucleotide&term=NM_004154) | [Hs.16362](http://www.ncbi.nlm.nih.gov/entrez/query.fcgi?cmd=search&db=unigene&term=Hs.16362&dopt=unigene) | pyrimidinergic receptor P2Y, G-protein coupled, 6 | 0.619286 |
| ZNF85 | [NM_003429](http://www.ncbi.nlm.nih.gov/entrez/query.fcgi?cmd=search&db=nucleotide&term=NM_003429) | [Hs.37138](http://www.ncbi.nlm.nih.gov/entrez/query.fcgi?cmd=search&db=unigene&term=Hs.37138&dopt=unigene) | zinc finger protein 85 (HPF4, HTF1) | 0.615009 |
| RNGTT | [NM_003800](http://www.ncbi.nlm.nih.gov/entrez/query.fcgi?cmd=search&db=nucleotide&term=NM_003800) | [Hs.127219](http://www.ncbi.nlm.nih.gov/entrez/query.fcgi?cmd=search&db=unigene&term=Hs.127219&dopt=unigene) | RNA guanylyltransferase and 5'-phosphatase | 0.613018 |
| CABYR | [NM_012189](http://www.ncbi.nlm.nih.gov/entrez/query.fcgi?cmd=search&db=nucleotide&term=NM_012189) | [Hs.511983](http://www.ncbi.nlm.nih.gov/entrez/query.fcgi?cmd=search&db=unigene&term=Hs.511983&dopt=unigene) | calcium binding tyrosine-(Y)-phosphorylation regulated (fibrousheathin 2) | 0.611607 |
| TBPIP | [BE964655](http://www.ncbi.nlm.nih.gov/entrez/query.fcgi?cmd=search&db=nucleotide&term=BE964655) | [Hs.279032](http://www.ncbi.nlm.nih.gov/entrez/query.fcgi?cmd=search&db=unigene&term=Hs.279032&dopt=unigene) | NA | 0.601713 |
| NPR1 | [NM_000906](http://www.ncbi.nlm.nih.gov/entrez/query.fcgi?cmd=search&db=nucleotide&term=NM_000906) | [Hs.490330](http://www.ncbi.nlm.nih.gov/entrez/query.fcgi?cmd=search&db=unigene&term=Hs.490330&dopt=unigene) | natriuretic peptide receptor A/guanylate cyclase A (atrionatriuretic peptide receptor A) | 0.59293 |
| DAPP1 | [NM_014395](http://www.ncbi.nlm.nih.gov/entrez/query.fcgi?cmd=search&db=nucleotide&term=NM_014395) | [Hs.436271](http://www.ncbi.nlm.nih.gov/entrez/query.fcgi?cmd=search&db=unigene&term=Hs.436271&dopt=unigene) | dual adaptor of phosphotyrosine and 3-phosphoinositides | 0.591941 |
| MELK | [NM_014791](http://www.ncbi.nlm.nih.gov/entrez/query.fcgi?cmd=search&db=nucleotide&term=NM_014791) | [Hs.184339](http://www.ncbi.nlm.nih.gov/entrez/query.fcgi?cmd=search&db=unigene&term=Hs.184339&dopt=unigene) | maternal embryonic leucine zipper kinase | 0.589271 |
| RAD51AP1 | [BE966146](http://www.ncbi.nlm.nih.gov/entrez/query.fcgi?cmd=search&db=nucleotide&term=BE966146) | [Hs.504550](http://www.ncbi.nlm.nih.gov/entrez/query.fcgi?cmd=search&db=unigene&term=Hs.504550&dopt=unigene) | RAD51 associated protein 1 | 0.584931 |
| TRIP13 | [NM_004237](http://www.ncbi.nlm.nih.gov/entrez/query.fcgi?cmd=search&db=nucleotide&term=NM_004237) | [Hs.436187](http://www.ncbi.nlm.nih.gov/entrez/query.fcgi?cmd=search&db=unigene&term=Hs.436187&dopt=unigene) | thyroid hormone receptor interactor 13 | 0.582128 |
| CCNE2 | [NM_004702](http://www.ncbi.nlm.nih.gov/entrez/query.fcgi?cmd=search&db=nucleotide&term=NM_004702) | [Hs.408658](http://www.ncbi.nlm.nih.gov/entrez/query.fcgi?cmd=search&db=unigene&term=Hs.408658&dopt=unigene) | cyclin E2 | 0.572964 |
| ZNF669 | [NM_024804](http://www.ncbi.nlm.nih.gov/entrez/query.fcgi?cmd=search&db=nucleotide&term=NM_024804) | [Hs.163754](http://www.ncbi.nlm.nih.gov/entrez/query.fcgi?cmd=search&db=unigene&term=Hs.163754&dopt=unigene) | zinc finger protein 669 | 0.567312 |
| CHRNB2 | [NM_000748](http://www.ncbi.nlm.nih.gov/entrez/query.fcgi?cmd=search&db=nucleotide&term=NM_000748) | [Hs.2306](http://www.ncbi.nlm.nih.gov/entrez/query.fcgi?cmd=search&db=unigene&term=Hs.2306&dopt=unigene) | cholinergic receptor, nicotinic, beta polypeptide 2 (neuronal) | 0.558279 |
| KNTC1 | [NM_014708](http://www.ncbi.nlm.nih.gov/entrez/query.fcgi?cmd=search&db=nucleotide&term=NM_014708) | [Hs.300559](http://www.ncbi.nlm.nih.gov/entrez/query.fcgi?cmd=search&db=unigene&term=Hs.300559&dopt=unigene) | kinetochore associated 1 | 0.556752 |
| IFNAR2 | [NM_000874](http://www.ncbi.nlm.nih.gov/entrez/query.fcgi?cmd=search&db=nucleotide&term=NM_000874) | [Hs.549042](http://www.ncbi.nlm.nih.gov/entrez/query.fcgi?cmd=search&db=unigene&term=Hs.549042&dopt=unigene) | interferon (alpha, beta and omega) receptor 2 | 0.542515 |
| LMNB1 | [NM_005573](http://www.ncbi.nlm.nih.gov/entrez/query.fcgi?cmd=search&db=nucleotide&term=NM_005573) | [Hs.89497](http://www.ncbi.nlm.nih.gov/entrez/query.fcgi?cmd=search&db=unigene&term=Hs.89497&dopt=unigene) | lamin B1 | 0.539327 |
| TDP1 | [NM_018319](http://www.ncbi.nlm.nih.gov/entrez/query.fcgi?cmd=search&db=nucleotide&term=NM_018319) | [Hs.209945](http://www.ncbi.nlm.nih.gov/entrez/query.fcgi?cmd=search&db=unigene&term=Hs.209945&dopt=unigene) | tyrosyl-DNA phosphodiesterase 1 | 0.533353 |
| 38596 | [AA702163](http://www.ncbi.nlm.nih.gov/entrez/query.fcgi?cmd=search&db=nucleotide&term=AA702163) | [Hs.283743](http://www.ncbi.nlm.nih.gov/entrez/query.fcgi?cmd=search&db=unigene&term=Hs.283743&dopt=unigene) | septin 5 | 0.530598 |
| MCM2 | [NM_004526](http://www.ncbi.nlm.nih.gov/entrez/query.fcgi?cmd=search&db=nucleotide&term=NM_004526) | [Hs.477481](http://www.ncbi.nlm.nih.gov/entrez/query.fcgi?cmd=search&db=unigene&term=Hs.477481&dopt=unigene) | MCM2 minichromosome maintenance deficient 2, mitotin (S. cerevisiae) | 0.526137 |
| PRC1 | [NM_003981](http://www.ncbi.nlm.nih.gov/entrez/query.fcgi?cmd=search&db=nucleotide&term=NM_003981) | [Hs.459362](http://www.ncbi.nlm.nih.gov/entrez/query.fcgi?cmd=search&db=unigene&term=Hs.459362&dopt=unigene) | protein regulator of cytokinesis 1 | 0.525493 |
| PPP2R5D | [NM_006245](http://www.ncbi.nlm.nih.gov/entrez/query.fcgi?cmd=search&db=nucleotide&term=NM_006245) | [Hs.533308](http://www.ncbi.nlm.nih.gov/entrez/query.fcgi?cmd=search&db=unigene&term=Hs.533308&dopt=unigene) | protein phosphatase 2, regulatory subunit B (B56), delta isoform | 0.524502 |
| TEAD4 | [NM_003213](http://www.ncbi.nlm.nih.gov/entrez/query.fcgi?cmd=search&db=nucleotide&term=NM_003213) | [Hs.94865](http://www.ncbi.nlm.nih.gov/entrez/query.fcgi?cmd=search&db=unigene&term=Hs.94865&dopt=unigene) | TEA domain family member 4 | 0.51957 |
| PPARD | [NM_006238](http://www.ncbi.nlm.nih.gov/entrez/query.fcgi?cmd=search&db=nucleotide&term=NM_006238) | [Hs.485196](http://www.ncbi.nlm.nih.gov/entrez/query.fcgi?cmd=search&db=unigene&term=Hs.485196&dopt=unigene) | peroxisome proliferative activated receptor, delta | 0.519112 |
| ERN2 | [AI732416](http://www.ncbi.nlm.nih.gov/entrez/query.fcgi?cmd=search&db=nucleotide&term=AI732416) | [Hs.528301](http://www.ncbi.nlm.nih.gov/entrez/query.fcgi?cmd=search&db=unigene&term=Hs.528301&dopt=unigene) | endoplasmic reticulum to nucleus signalling 2 | 0.516475 |
| C10orf3 | [NM_018131](http://www.ncbi.nlm.nih.gov/entrez/query.fcgi?cmd=search&db=nucleotide&term=NM_018131) | [Hs.14559](http://www.ncbi.nlm.nih.gov/entrez/query.fcgi?cmd=search&db=unigene&term=Hs.14559&dopt=unigene) | chromosome 10 open reading frame 3 | 0.507976 |
| HFE | [AF144241](http://www.ncbi.nlm.nih.gov/entrez/query.fcgi?cmd=search&db=nucleotide&term=AF144241) | [Hs.233325](http://www.ncbi.nlm.nih.gov/entrez/query.fcgi?cmd=search&db=unigene&term=Hs.233325&dopt=unigene) | hemochromatosis | 0.507621 |
| RPL23AP13 | [NM_020217](http://www.ncbi.nlm.nih.gov/entrez/query.fcgi?cmd=search&db=nucleotide&term=NM_020217) | [Hs.534472](http://www.ncbi.nlm.nih.gov/entrez/query.fcgi?cmd=search&db=unigene&term=Hs.534472&dopt=unigene) | NA | 0.507549 |
| TACC3 | [NM_006342](http://www.ncbi.nlm.nih.gov/entrez/query.fcgi?cmd=search&db=nucleotide&term=NM_006342) | [Hs.104019](http://www.ncbi.nlm.nih.gov/entrez/query.fcgi?cmd=search&db=unigene&term=Hs.104019&dopt=unigene) | transforming, acidic coiled-coil containing protein 3 | 0.506786 |
| RRM2 | [BE966236](http://www.ncbi.nlm.nih.gov/entrez/query.fcgi?cmd=search&db=nucleotide&term=BE966236) | [Hs.226390](http://www.ncbi.nlm.nih.gov/entrez/query.fcgi?cmd=search&db=unigene&term=Hs.226390&dopt=unigene) | ribonucleotide reductase M2 polypeptide | 0.503332 |
| GMEB2 | [AL133646](http://www.ncbi.nlm.nih.gov/entrez/query.fcgi?cmd=search&db=nucleotide&term=AL133646) | [Hs.473286](http://www.ncbi.nlm.nih.gov/entrez/query.fcgi?cmd=search&db=unigene&term=Hs.473286&dopt=unigene) | glucocorticoid modulatory element binding protein 2 | 0.503213 |
| CCNB2 | [NM_004701](http://www.ncbi.nlm.nih.gov/entrez/query.fcgi?cmd=search&db=nucleotide&term=NM_004701) | [Hs.194698](http://www.ncbi.nlm.nih.gov/entrez/query.fcgi?cmd=search&db=unigene&term=Hs.194698&dopt=unigene) | cyclin B2 | 0.502737 |
| LILRA4 | [AF041261](http://www.ncbi.nlm.nih.gov/entrez/query.fcgi?cmd=search&db=nucleotide&term=AF041261) | [Hs.406708](http://www.ncbi.nlm.nih.gov/entrez/query.fcgi?cmd=search&db=unigene&term=Hs.406708&dopt=unigene) | leukocyte immunoglobulin-like receptor, subfamily A (with TM domain), member 4 | 0.501853 |
