# Supplementary material for: Overexpression of POLQ Confers a Poor Prognosis in Early Breast Cancer Patients
Source: Oncotarget. 2010 Jul 9;1(3):175–84. doi: 10.18632/oncotarget.124 (PMC2917771; doi:10.18632/oncotarget.124)
Supplement: Supplementary Table 6 [file oncotarget-01-175-s006.doc]

Supplementary Table 6. Results of the multivariate analyses performed on the data from Series 1 after inclusion of CCNE2 and the expression profiles previously described. Data summarised in Fig 4A.

Multivariate analysis including CCNE2:

|  |  |  |  |  | 95.0% CI for HR | |
| --- | --- | --- | --- | --- | --- | --- |
|  | B | SE | p | Hazard Ratio | Lower | Upper |
| POLQ2 score | 1.5039 | 0.7000 | 0.0317 | 4.4990 | 1.1410 | 17.7392 |
| Age | 0.0031 | 0.0173 | 0.8570 | 1.0031 | 0.9697 | 1.0377 |
| Grade | -0.1982 | 0.2871 | 0.4900 | 0.8202 | 0.4672 | 1.4398 |
| Tumour Size | 0.2264 | 0.1039 | 0.0293 | 1.2540 | 1.0231 | 1.5372 |
| ER status | -0.5609 | 0.5985 | 0.3487 | 0.5707 | 0.1766 | 1.8444 |
| Tamoxifen | 0.2888 | 0.5242 | 0.5816 | 1.3349 | 0.4778 | 3.7293 |
| Nodal status | 0.0038 | 0.0762 | 0.9601 | 1.0038 | 0.8646 | 1.1655 |
| CCNE2 score | 1.7018 | 0.7399 | 0.0214 | 5.4836 | 1.2861 | 23.3803 |

Backward Likelihood Reduced model :

|  |  |  |  |  | 95.0% CI for HR | |
| --- | --- | --- | --- | --- | --- | --- |
|  | B | SE | p | Hazard Ratio | Lower | Upper |
| Tumour Size | 0.2166 | 0.0897 | 0.0158 | 1.2418 | 1.0416 | 1.4805 |
| POLQ2 score | 1.4203 | 0.6364 | 0.0256 | 4.1383 | 1.1889 | 14.4046 |
| CCNE2 score | 1.6191 | 0.6815 | 0.0175 | 5.0488 | 1.3278 | 19.1974 |

Multivariate analysis including GGI:

|  |  |  |  |  | 95.0% CI for HR | |
| --- | --- | --- | --- | --- | --- | --- |
|  | B | SE | p | Hazard Ratio | Lower | Upper |
| POLQ2 score | 1.2387 | 0.6309 | 0.0496 | 3.4510 | 1.0021 | 11.8847 |
| Age | 0.0152 | 0.0159 | 0.3380 | 1.0153 | 0.9842 | 1.0474 |
| Tumour Size | 0.1754 | 0.0869 | 0.0435 | 1.1917 | 1.0052 | 1.4129 |
| ER status | -0.1149 | 0.4885 | 0.8140 | 0.8915 | 0.3422 | 2.3222 |
| Tamoxifen | -0.1936 | 0.4016 | 0.6297 | 0.8240 | 0.3751 | 1.8102 |
| Nodal status | 0.0172 | 0.0634 | 0.7861 | 1.0173 | 0.8985 | 1.1519 |
| GGI score | 0.2418 | 0.1488 | 0.1042 | 1.2736 | 0.9514 | 1.7049 |

Backward Likelihood Reduced model :

|  |  |  |  |  | 95.0% CI for HR | |
| --- | --- | --- | --- | --- | --- | --- |
|  | B | SE | p | Hazard Ratio | Lower | Upper |
| Tumour Size | 0.1799 | 0.0805 | 0.0254 | 1.1971 | 1.0224 | 1.4017 |
| POLQ2 score | 1.1876 | 0.6296 | 0.0593 | 3.2791 | 0.9547 | 11.2629 |
| GGI score | 0.2482 | 0.1386 | 0.0733 | 1.2817 | 0.9769 | 1.6817 |

Multivariate analysis including 76 gene signature:

|  |  |  |  |  | 95.0% CI for HR | |
| --- | --- | --- | --- | --- | --- | --- |
|  | B | SE | p | Hazard Ratio | Lower | Upper |
| POLQ2 score | 1.6378 | 0.6400 | 0.0105 | 5.1439 | 1.4673 | 18.0330 |
| Age | 0.0009 | 0.0178 | 0.9589 | 1.0009 | 0.9666 | 1.0365 |
| Grade | -0.2085 | 0.2675 | 0.4356 | 0.8118 | 0.4806 | 1.3712 |
| Tumour Size | 0.2199 | 0.1081 | 0.0420 | 1.2460 | 1.0080 | 1.5402 |
| ER status | -1.9410 | 0.7471 | 0.0094 | 0.1436 | 0.0332 | 0.6208 |
| Tamoxifen | 0.2904 | 0.5109 | 0.5698 | 1.3369 | 0.4912 | 3.6391 |
| Nodal status | 0.0123 | 0.0816 | 0.8800 | 1.0124 | 0.8628 | 1.1879 |
| 76 gene | 2.6936 | 0.9077 | 0.0030 | 14.7851 | 2.4955 | 87.5972 |

Backward Likelihood Reduced model :

|  |  |  |  |  | 95.0% CI for HR | |
| --- | --- | --- | --- | --- | --- | --- |
|  | B | SE | p | Hazard Ratio | Lower | Upper |
| Tumour Size | 0.2182 | 0.0912 | 0.0167 | 1.2438 | 1.0402 | 1.4872 |
| ER status | -1.5408 | 0.5678 | 0.0067 | 0.2142 | 0.0704 | 0.6519 |
| POLQ2 score | 1.4943 | 0.5920 | 0.0116 | 4.4562 | 1.3964 | 14.2203 |
| 76 gene | 2.5696 | 0.8865 | 0.0038 | 13.0602 | 2.2979 | 74.2299 |

Multivariate analysis including 70 gene signature:

|  |  |  |  |  | 95.0% CI for HR | |
| --- | --- | --- | --- | --- | --- | --- |
|  | B | SE | p | Hazard Ratio | Lower | Upper |
| POLQ2 score | 1.7584 | 0.7558 | 0.0200 | 5.8031 | 1.3193 | 25.5260 |
| Age | 0.0065 | 0.0175 | 0.7090 | 1.0065 | 0.9727 | 1.0416 |
| Grade | -0.0815 | 0.2752 | 0.7672 | 0.9218 | 0.5375 | 1.5807 |
| Tumour Size | 0.2409 | 0.1030 | 0.0194 | 1.2724 | 1.0397 | 1.5572 |
| ER status | -0.5224 | 0.5990 | 0.3831 | 0.5931 | 0.1833 | 1.9184 |
| Tamoxifen | 0.2063 | 0.5179 | 0.6903 | 1.2292 | 0.4455 | 3.3917 |
| Nodal status | -0.0221 | 0.0752 | 0.7694 | 0.9782 | 0.8441 | 1.1336 |
| 70 gene | 0.6413 | 0.7805 | 0.4113 | 1.8989 | 0.4113 | 8.7671 |
